# Association between Sexual Satisfaction and Depression and Anxiety in Adolescents and Young Adults

**DOI:** 10.3390/ijerph17030841

**Published:** 2020-01-29

**Authors:** Rodrigo J. Carcedo, Noelia Fernández-Rouco, Andrés A. Fernández-Fuertes, José Luis Martínez-Álvarez

**Affiliations:** 1 Department of Developmental and Educational Psychology, University of Salamanca, 37005 Salamanca, Spain; rcarcedo@usal.es (R.J.C.); maral@usal.es (J.L.M.-Á.); 2 Department of Education, University of Cantabria, 39005 Santander, Spain; fernandezaa@unican.es

**Keywords:** sexual satisfaction, mental health, romantic relationships, adolescents, young adults

## Abstract

The role of sexual satisfaction in adolescents and young adults’ mental health has not been thoroughly investigated. The aim of this work is to study differences in sexual satisfaction and mental health (anxiety and depression) based on romantic relationship status (having a partner vs. not having one) and gender. Likewise, the association between sexual satisfaction and mental health and the moderating effect of romantic relationship status and gender was addressed in this research. A total of 1682 Spanish adolescents (14–17) and young adults (18–29) agreed to participate in this cross-sectional investigation. Two-factor ANOVA and MANOVA, and hierarchical regression models were utilized in this study. In general, results showed more difficulties in sexual satisfaction and mental health for those not in a current relationship and for women. Additionally, higher levels of sexual satisfaction was associated with lower levels of anxiety for adolescents and lower levels of depression for young adults. These associations were stronger for those in a current relationship. This study highlights the importance of sexual satisfaction as a modifying factor against mental health problems, especially in the context of a current romantic relationship.

## 1. Introduction

Traditionally, the study of romantic and sexual awakening has been linked to the concept of risk, danger or threat to the healthy development and psychological adjustment of adolescents and young adults [1,2]. More recently, however, studies have looked at important contributions made in the field of sexual development in regard to physical and mental health, well-being, and quality of life; in fact, a normative and positive view of adolescent sexuality has now been proposed [3,4,5,6]. 

Indeed, sexual satisfaction is not merely considered to be one dimension of sexual health, but as a sexual right and an outcome of sexual well-being and global health as well [7,8]. It has commonly been conceptualized in terms of positive affect [9] including “the degree to which an individual is satisfied or happy with the sexual aspect of his or her relationship“ [10] (p. 236) and “an affective response arising from one’s subjective evaluation of the positive and negative dimensions associated with one’s sexual relationship” [11] (p. 268), a definition stemming from the Interpersonal Exchange Model of Sexual Satisfaction. All these definitions assume that feeling satisfied is a subjective experience. Lay definitions of sexual satisfaction have similarly focused on positive outcomes or rewards, rather than on the absence of costs, as would be considered from the perspective of a social exchange model [12]. 

The main objective of this work is to explore the association between sexual satisfaction and mental health and the moderating effect that romantic relationship status and gender have on this relationship. This analysis was carried out at two developmental stages involving two different developmental tasks pertaining to sexual and romantic life. That is, an exploration of these relationships throughout adolescence, and the preparation of future family roles as young adults [13]. Additionally, differences in sexual satisfaction and mental health (anxiety and depression) based on romantic relationship status and gender were also analyzed. 

Differences in sexual satisfaction and mental health have been reported as a function of relationship status and gender. A large portion of adolescents and young adult’s sexual activities takes place within the context of romantic relationships [1] although some take place outside such relationships [14]. An important line of research focuses on certain characteristics identified in adolescent and young adult romantic relationships and the associations they have with sexual satisfaction and mental health. For example, a satisfactory relationship and relational processes (e.g., greater intimacy, good dyadic adjustment, communication, and partner support) have repeatedly been found to be associated with sexual satisfaction [15] and mental health [1]. This current research is part of a larger group of studies that have addressed differences in sexual satisfaction and mental health between those currently in a relationship and those who are not. 

In this sense, sexual satisfaction is identified as a crucial factor in the differences identified among people in a relationship and those who are single, presumably prompting those in a relationship to operate at a higher level of fulfillment [16]. Furthermore, higher levels of sexual satisfaction have been found among adolescents in a romantic relationship [17] as well as among young adults and adults [18,19]. Being in a romantic relationship increases the likelihood that sexual activity will occur at any age and also is a key context for understanding access to sexual activity in adolescence. Positive sexual experiences, which imply greater sexual satisfaction, are likewise encouraged within the context of affective relationships [1].

With respect to mental health, although extensive research has been conducted on anxiety and depression among adults, little is known about profiles of depression and anxiety throughout the life cycle. In addition, the role that having a romantic partner plays in mental health may vary depending on the life stage. Most of the studies on young adults’ population shows that those in romantic relationships tend to exhibit better mental health than those who are single [20].

Notwithstanding, the small body of work on intimate relationships and mental health in adolescence has produced less consistent results. While some studies find that these relationships enhance adolescents’ emotional well-being [21] and claim that high-quality relationships are linked with lower levels of depression [22] and anxiety [23], studies indicate quite the opposite, that depression levels [24] and substance problems [25] actually increase. This is consistent with the few longitudinal studies that have been done, which yielded increased depressive symptoms over time among adolescents who were in, or became involved in a romantic relationship [26,27]. Thus, the association between being in a romantic relationship and adolescent mental health remains unclear.

To sum up, being in a romantic relationship could be a protective factor to maintaining good mental health [28] especially for adults and young adults, although this relationship is yet unclear among adolescents. There is more evidence that being in a romantic relationship is associated with higher levels of sexual satisfaction in both adolescence and young adulthood than otherwise.

Gender differences in sexual satisfaction during adolescence and young adulthood have also been observed. Correspondingly, men generally report higher consistency of sexual activity than women in studies of adolescent and young adult sexuality [29], and young men report markedly more positive emotions after sex, while young women report markedly more negative ones [30]. However, no differences in sexual satisfaction between genders have also been found in young adults and adults [31,32].

Results in terms of gender could be also contradictory due to the characteristics of instruments assessing sexual satisfaction: men are likely to show higher levels of sexual satisfaction than women if the questionnaire is focused on physical aspects, and women are likely to express sexual satisfaction if the instrument has more items related to relational aspects [15].

Gender differences are likewise a common theme in the study of mental health. It is well established that gender differences exist in rates of depression and anxiety disorders, with women more likely to experience these disorders than men [33,34,35,36]. Women are more likely than men to experience emotional problems and to report a history of non-suicidal self-injury [37]. Men, on the other hand, are more likely to exhibit conduct or behavioral problems, both in adolescence [38] and young adulthood [39]. Accordingly, adolescent women were significantly more likely than men to correctly label depression, yet sex differences were not found in relation to social anxiety [40].

Although the scientific literature shows great interest in the study of the relationship [41,42,43] between sexual satisfaction [15] and mental health, little research exists regarding this association among young people, especially adolescents. In fact, a majority of the studies were conducted with adult participants, and many of them in the context of clinical samples [44,45,46]. 

A positive association between sexual satisfaction and mental health or well-being was identified in adult samples. In general, a depressed mood or certain depressive symptoms are associated with an increase of sexual dissatisfaction in the adult population [15]. Other studies have similarly shown the same positive association but focusing on the dark side of relationships: they claim that sexual dissatisfaction can be associated with depressed moods [42,43] and lower rates of general psychological well-being [47]. 

The association between sexual activity and well-being in adolescence remains a controversial issue; other studies however, have similarly found a positive relationship between sexual activity and depression during adolescence [48,49]. In fact, research in adolescence has paved the way for the study of possible moderating factors in this relationship, despite the significant lack of studies addressing the relationship between sexual satisfaction and mental health and its possible moderators in both adolescents and young adults.

An exception is the work carried out in the USA by Vasilenko [49], which used a general sample of adolescents and young adults. In this study, sexual behavior of adolescents was found to be associated with symptoms of depression, especially among girls. In addition, sex with a non-relationship partner was associated with an increase in depressive symptoms for adolescent girls, but not for adolescent boys. As the author of this research explains, girls are more likely to be influenced by socio-cultural norms regarding sexual behaviour, with sexual intercourse being widely regarded as "inappropriate" for girls but not for boys [49]. Among young people, that association became weaker or nonexistent for both genders and different relationship statuses, maybe because at this developmental stage sexual behavior is more normative as they got older. However, in a study with college students from the USA, involvement in a romantic relationship, compared to being single, was found to be associated with fewer depressive symptoms, yet only for women [50]. It is reasonable to think that sexual satisfaction may be more important for the mental health and well-being of those in a current relationship and those whose gender role suffers more sociocultural pressures, as has been found in the case of women, although current evidence suggests otherwise. 

In summary, the association between sexual satisfaction and mental health or well-being in adolescents and young adults is complex, and important aspects such as gender and current romantic relationship status may play a role. There is a lack of scientific knowledge on the subject, especially in adolescence, and research must be conducted in different cultures as social and cultural factors come into play. As a consequence of the aforementioned, the present research furthers scientific knowledge investigating the differences in gender and relationship status in sexual satisfaction and mental health, in addition to the possible association between sexual satisfaction and mental health moderated by gender and relationship status in both adolescents and young adults. 

As previously stated, the empirical literature regarding the role of relationship status and gender in sexual satisfaction and mental health remains unclear for adolescents and young adults. In view of these considerations, two main research questions were established in this study: (1) Are there any differences in sexual satisfaction and mental health (anxiety and depression) with regard to relationship status (having a partner vs. not having one) and gender (men vs. women) for adolescents and young adults? and (2) Do relationship status and gender moderate the link between sexual satisfaction and mental health (anxiety and depression) for adolescents and young adults?

## 2. Materials and Methods 

### 2.1. Participants

The final sample used for analysis was comprised of 1682 heterosexual adolescents and young people: 732 (43.50%) were men and 950 (56.50%) were women; all between 14 and 29 years old (M = 17.97; SD = 2.71); that is 809 adolescents (42.89% were boys and 57.11% were girls) between 14 and 17 years old (M = 15.86; SD = 1.01) and 873 young adults (44.10% were men and 55.90% were women) between 18 and 29 years old (M = 19.92; SD = 2.31); 887 (55.50%) were secondary school students and 710 (44.50%) were university students; 1075 (63.91%) participants were in a current serious exclusive romantic relationship of one month or more; the average duration of the relationship was 16.12 months (SD = 19.09). Regarding the moderators (gender and partner status) and the relationship variables in this study, the adolescent group exhibited, as expected, a lower frequency of participants in a current romantic relationship (χ^2^(1) = 46.28, *p* < 0.001) and relationships of shorter duration (t(525.88) = −13.05, *p* < 0.001) than was the case with the young adult group. No gender differences were found between adolescents and young adults (χ^2^(1) = 0.25, *p* > 0.05).

We used the same time frame that Rogers et al. used in their work on the state of couple relationships and cohabitation (Project READY (Researching Emerging Adults Developmental Years) for young adults (18 to 29 years old) [13]. The youngest age commonly found in young adult studies is 18 years old, which corresponds to the legal age of adulthood and the age at which a young person transitions from high school to college in Spain. By contrast, adolescent studies reveal ample variability of time frames based on different criteria (e.g., biological, social processes, etc.) [51]. For this reason, we set up the cut-off point between adolescence and young adulthood at 18 years old. 

Participants were recruited from universities and secondary schools from five different Spanish provinces from the Extremadura and Castile and Leon regions. They were given the questionnaire once they signed the consent form. A total of 3 students rejected participation or withdrew during the application process (0.17% of the original sample, *n* = 1770); 52 questionnaires presented incomplete answers (2.94%); 5 participants considered themselves to be in a serious romantic relationship but the duration was less than a month (0.28%), and 28 identified themselves as non-heterosexual (1.58%). None of these participants was part of the final sample. 

### 2.2. Design and Procedure

A cross-sectional (one moment application study) and correlational (association between variables where causation may not be inferred) design was used to examine the relationship between sexual satisfaction and the mental health of adolescents and young adults (i.e., levels of anxiety and depression), along with the moderating effect of relationship status and gender.

The procedure was as follows: a list of all secondary schools and universities in each province was generated and from that list an Excel program was used to randomly select seven secondary schools and five faculties from two universities to be invited to participate in the study. From those contacted, five secondary schools and all the university faculties agreed to take part in the research. Once the institutions agreed to participate, participants and their families, the latter when necessary (i.e., minors), were contacted by the head of the school’s administration and implementation of the program was organized, including laying out dates for the implementation and the various conditions. In the case of underage participants, a questionnaire was sent from the schools which included an explanation of the nature and main characteristics of the study and requested written informed consent from the parents. With respect to of-age participants, the faculties informed the students about the study and one teacher of the class informed the students. A date for the application was agreed upon and written informed consent was given at the time of application. In all cases, the study was implemented with paper-pencil questionnaires. All participants agreed to collaborate voluntarily, without material incentive. This study was carried out with the approval of the Bioethics Committee of the University (ethical code) and all subjects gave written informed consent in accordance with the Declaration of Helsinki. Data collection was always carried out by at least one member of the research team who reminded participants of the aim of the study and outlined the details of the questionnaires. Participants were informed that their answers were anonymous and confidential and that they could refuse to participate or withdraw from the study at any time. About 25 minutes were needed to complete the questionnaire. Only those questionnaires that were fully completed were analyzed.

### 2.3. Measures

#### 2.3.1. Predictor Variable: Sexual Satisfaction 

The subscale of sexual satisfaction of the Multidimensional Sexual Self-Concept Questionnaire (MSSCQ) was used to measure this construct [52]. A total of five items (e.g., “I am satisfied with the way my sexual needs are currently being met”) was each scored on a 5-point Likert-type scale that ranged from 1 (i.e., “Not at all characteristic of me”) to 5 (i.e., “Very characteristic of me”). An initial translation was made by authors from English to Spanish, and then a second translation was made back to English. After reaching an agreement, a panel of two English/Spanish translators assessed the Spanish translations for quality, and three English-speaking sexuality expert psychologists assessed the final translation. The total sexual satisfaction score regarding the last month was obtained by adding up the individual scores of each item and dividing them by the number of items answered, with possible scores ranging from 1 (i.e., “Totally dissatisfied”) to 5 (i.e., “Totally satisfied”). Cronbach’s alpha in this study was 0.95.

#### 2.3.2. Criterion Variables: Anxiety and Depression 

The anxiety and depression subscales of the Symptom Checklist (SCL-90-R) were used to assess anxiety and depression [53]. Twenty-three items were scored: ten items for anxiety and thirteen for depression; for each item, the person was asked to rate the emotion severity level experienced over the past several weeks. Responses were recorded on a 5-point Likert-type scale ranging from 1 (i.e., “Not at all”) to 5 (i.e., “Extremely”). A total score was obtained for each subscale by adding up the individual scores and dividing them by the number of items answered, with possible scores ranging from 1 (i.e., “No depression or anxiety at all”) to 5 (i.e., “High levels of depression or anxiety”). In this study, Cronbach’s alpha for anxiety was 0.87 and Cronbach’s alpha for depression was 0.89.

#### 2.3.3. Moderating Variables: Relationship Status and Gender

Relationship status was coded as 0 for participants not currently in a romantic relationship or in a relationship which lasted less than one month, and 1 for participants currently in a romantic relationship lasting one month or longer; in addition, it was important that the relationship was seen as both serious and romantic. Gender was coded as 0 for men and 1 for women.

### 2.4. Data Analysis

A two-factor ANOVA was used to analyze the differences in sexual satisfaction between gender and relationship status. Additionally, a two-factor MANOVA was run to recognize gender and relationship status differences in depression and anxiety.

A series of hierarchical regression models using the macro PROCESS 3.2. for SPSS [54] were conducted to study the predictors of each mental measure for adolescents and young adults. Main effects were entered at the first step, two-way interactions at the second, and the three-way interaction at the third one. The interaction model was selected when the increment in R^2^ in the step was significant due to the inclusion of the interaction terms into the regression equation. In this case, the significant highest-order interaction model was chosen. PROCESS’s model numbers 1 and 2 for two-way interactions (also known as simple moderation), and 3 for the three-way interaction (also named moderated moderation) were used. Additionally, 95% confidence intervals (CI) were calculated based on 5000 bootstrap samples. For all analyses, statistical significance was defined as *p* < 0.05. All the statistical analyses were conducted using the IBM SPSS 23 package (IBM Corp., Armonk, NY, USA). Regarding missing data, a listwise deletion of cases was used. Therefore, those participants who lacked data on any of the analyzed variables were removed from analysis (*n* = 52).

## 3. Results

The first research question of this study addresses differences in sexual satisfaction and mental health with respect to relationship status and gender for adolescents and young adults. A 2 (gender) × 2 (relationship status) ANOVA was conducted to examine differences in sexual satisfaction. In addition, a 2 (gender) × 2 (relationship status) MANOVA was used to identify differences in mental health measures when tested together (anxiety and depression) and follow-up ANOVAs were utilized to study differences in anxiety and depression when tested separately (see Table 1).

Regarding adolescents, ANOVA revealed a significant main effect of relationship status for sexual satisfaction, with lower levels of sexual satisfaction for those not in a current romantic relationship than those in a current romantic relationship. There was no significant main effect for gender nor was there an interaction between gender and relationship status. In addition, MANOVA found a significant main effect of gender for mental health (Wilks’s lambda = 0.96; *F*(2, 804) = 14.94, *p* < 0.001, η^2^_p_ = 0.036), whereas neither the main effect of relationship status (Wilks’s lambda = 0.99; *F*(2, 804) = 2.21, *p* > 0.05, η^2^_p_ = 0.005), nor the interaction of gender and relationship status (Wilks’s lambda = 0.99; *F*(2, 804) = 1.12, *p* > 0.05, η^2^_p_ = 0.003), was significant. Follow-up ANOVAs showed gender effects for both anxiety and depression. Adolescent girls presented higher levels of anxiety and depression than did adolescent boys (see Table 1).

Some similar results were found for young adults. Again, ANOVA revealed a significant main effect of relationship status for sexual satisfaction, with lower levels of sexual satisfaction for those not in a current romantic relationship than those in a current romantic relationship. No significant effects were observed for gender nor were they observed for the interaction between gender and relationship status. Addressing mental health, MANOVA not only found two significant main effects with respect to gender (Wilks’s lambda = 0.94; *F*(2, 868) = 27.79, *p* < 0.001, η^2^_p_ = 0.060) and relationship status (Wilks’s lambda = 0.99; *F*(2, 868) = 4.34, *p* < 0.05, η^2^_p_ = 0.010), but the interaction of gender and relationship status was also detected as significant (Wilks’s lambda = 0.99; *F*(2, 868) = 3.93, *p* < 0.05, η^2^_p_ = 0.009). Follow-up ANOVAs confirmed the significant main effects of gender and relationship status for both anxiety and depression, and also the gender × relationship status interaction exclusively for depression. Young adult women present higher levels of anxiety and depression than men, whereas greater scores in both variables are observed for those not in a romantic relationship in comparison with those in a current romantic relationship. In order to interpret the interaction, Bonferroni post-hoc multiple comparisons reveal significant differences in depression for young adult men (*p* < 0.001) in a relationship compared to those not in one, whereas no differences were found for women (*p* > 0.05). Men not in a current romantic relationship show higher levels of depression than those in one. Lastly, no interaction between gender and relationship status is found to be significant with respect to the anxiety variable.

**Figure 1:**
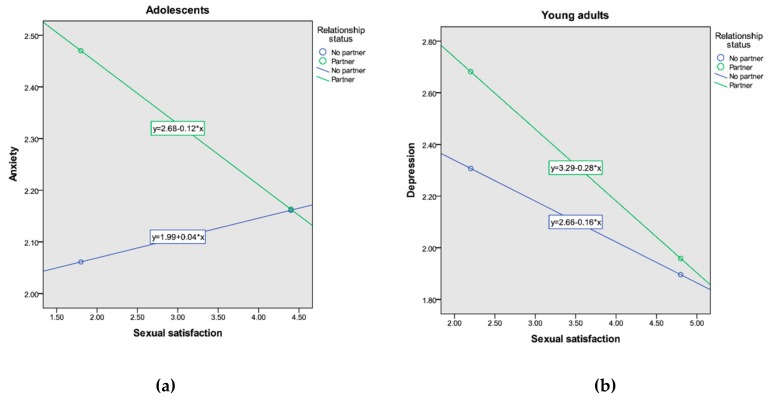
Sexual satisfaction × romantic relationship status interaction predicting mental health: (**a**) anxiety for adolescents; (**b**) depression for young adults.

The second research question prompted us to analyze the effect of sexual satisfaction with respect to the participants’ levels of anxiety and depression by studying the moderating role of gender and relationship status in adolescents and young adults. The interaction of sexual satisfaction and relationship status is significant as an explanation for anxiety among adolescents, and depression among young adults (see Table 2). The interpretation of the interaction indicated that lower levels of sexual satisfaction are associated with significantly higher levels of anxiety only for those adolescents in a current romantic relationship (*B* = −0.12, 95% CI (−0.23, 0.01)), but not for those not in a relationship (*B* = 0.04, 95% CI (−0.22, 0.07)) (see Figure 1a). Additionally, lower levels of sexual satisfaction are associated with significantly higher levels of depression for those young adults in a relationship (*B* = −0.28, *p* < 0.001, 95% CI (−0.22, −0.10)) and those not in a relationship (*B* = −0.16, 95% CI (−0.23, 0.01)), showing a greater association in the group with a romantic partner than in the group without (see Figure 1b). No other two-way interaction of sexual satisfaction with relationship status or gender was significant, nor was the three-way interaction (i.e., sexual satisfaction × gender × relationship status).

## 4. Discussion

Results regarding adolescents showed lower levels of sexual satisfaction in those not in a current romantic relationship, whereas higher levels of anxiety and depression were found among young women. Addressing young adults, those not in a romantic relationship presented lower levels of sexual satisfaction and greater anxiety and depression, whereas young women also exhibited higher levels of anxiety and depression. Furthermore, the interaction between romantic relationship status and gender was significant for depression. Young men without a current romantic partner exhibited higher levels of depression than young men in a current relationship, whereas no differences were found in young women.

In terms of sexual satisfaction, there is a lack of knowledge regarding aggregate assessments of individuals’ sexual satisfaction and whether or not satisfaction differs by demographics [44]. In this study, higher levels of sexual satisfaction were observed among those in a current romantic relationship during adolescence and adulthood. This finding is consistent with previous studies which found that participants not in a relationship exhibited lower levels of sexual satisfaction than those in a romantic relationship [55]. 

Combined with this result, no differences were observed between adolescent boys and girls and young adults. This finding is supported by previous studies [30,31], although other research has suggested the opposite [56]. Nevertheless, these conclusions are surely influenced by social and cultural factors, while there are no comparative studies regarding these variables among cultures, differences were found in regard to sexual development across countries and cultures [1]. Future studies should address this topic.

With regard to mental health, although extensive research has been conducted on anxiety and depression among adults, little is known about young adults and adolescents. The results of our study showed higher levels of anxiety and depression in girls throughout adolescence and on into young adulthood. In the same line, literature exists in which the results of our study are supported, and in which it is demonstrated that women tend to show higher levels of anxiety than men [57]. It is important to further investigate whether or not these gender differences pertaining to depression reflect a tendency for men to underreport depressive symptoms [58]. This research also found higher rates of anxiety and depression among those not in a relationship throughout young adulthood, but this is not true of those not in a relationship in adolescence. Previous research found that adolescents in romantic relationships demonstrate higher levels of depressive symptomatology than those who are not [49,59,60], though other studies observed that these relationships may enhance adolescents’ emotional well-being [21,22]. The effect of having been in a romantic relationship during adolescence or not has not yet yielded any conclusive results. In this sense, future research should focus on the specific characteristics of romantic relationships for those with a partner [22], and the interpersonal state and expectations associated with romantic relationships for those without a partner. 

Consistent with our findings, research on adult subjects, including young adults, demonstrate that those in romantic relationships tend to exhibit better mental health than those who are single [24,49,50]. In this vein, romantic relationships may play a modifying role in the mental health of young adults, as previously stated [28]. Based on the results of our study, this effect has a more significant role when related to depression among young men than among young women. Young men without a current romantic partner exhibit higher levels of depression than young men in a current relationship, whereas no differences were found in young women. The same result was identified among both single and married men and women [61]. From this vantage point, we speculate that women’s emotional state may be less affected by whether or not they have a partner, as women have traditionally been educated on how to manage their lives and daily routines, and also to develop close relationships that involve emotional expression outside of a romantic partnership. This reasoning is in line with other works addressing this issue of a double standard [62], which has implications for both men and women.

By way of conclusion, the above findings all support the importance attributed to romantic experiences as they pertain to the perception of sexual satisfaction and mental health and, therefore, positive development in adolescence and young adulthood [1,48]. 

With respect to the association between sexual satisfaction and mental health, and the moderating effect of romantic status and gender, the results of this study indicate that higher levels of sexual satisfaction are associated with lower levels of anxiety only for those in a romantic relationship throughout adolescence, and lower levels of depression for both current relationship statuses throughout young adulthood; this association being more prominent for those in a current relationship.

The relationship between sexual satisfaction and mental health, diversely, has been studied in the past, though these studies have expressly focused on adults, clinical samples, and women [45,63]. Within this context, mental health is seen as a predictor of sexual satisfaction [15,64], pointing out the effects that anxiety and depression generate in one’s sexual life. However, other studies found the opposite to be true, namely that sexual dissatisfaction is able to foretell mental health symptoms [42]. More specifically, sexual satisfaction is relevant to the mental health status of adolescents and young adults, and sexual problems can lead to anxiety and depression [43]. In fact, sexual satisfaction represents a key element in sexual health, romantic relationships, and mental health [65,66]. This study demonstrates that sexual satisfaction is significantly related to mental health. Furthermore, it was found that this association is only significant for those in a current relationship. This specific relationship is not only explained by the fact that most sexual relations between adolescents and young adults tend to occur in the context of romantic relationships [1], but it is also likely that those sexual relations with a romantic partner will be given greater importance.

Interestingly, a significant positive association between sexual satisfaction and anxiety in adolescence and between sexual satisfaction and depression in young adulthood was reported by those who were in a romantic relationship compared to those who were not. From a developmental perspective, adolescence signals the beginning of sexual behavior and sexual experimentation, while young adulthood is characterized by the consolidation of sexual relationships [67]. These developmental features create different social and personal pressures for adolescents and young adults. It could be speculated that low sexual satisfaction in the context of a romantic relationship during adolescence might generate more feelings of anxiety due to the fact that new experiences are surfacing and they result in increased feelings of insecurity, even though romantic relationships provide other important mental health benefits [50]. In the case of young adults, lack of sexual satisfaction can produce negative moods and emotions such as sadness, as a result of their perceived failure in this area of development.

Finally, gender did not play any moderating role in the association between sexual satisfaction and mental health. This finding is consistent with the lack of significant gender differences in sexual satisfaction found in this study. Although differences in mental health between men and women were identified, this study did not determine any significant differences in sexual satisfaction as a result of gender. Therefore, the subjective experience of sexual satisfaction and its implications on mental health is not significantly different for men and women.

These results support the development of competency and skill-based sex education aimed at promoting healthy and fulfilling romantic relationships. In short, this approach is consistent with the positive youth development model and the identified need to incorporate sex education as an integral component of this model [5]. In addition, although the association between being in a romantic relationship and mental health is bidirectional, more significant results are observed when mental health is the outcome and relationship status is the predictor, suggesting that mental health is more likely influenced by romantic relationships than the reverse. Therefore, it seems that it is more likely that improving romantic relationships would bring about improvements to mental health as opposed to the other way around [68]. Accordingly, a number of variables related to romantic relationships (especially for adolescents) is associated with the risk of attempted or completed suicide [69].

### 4.1. Limitations and Further Directions

The current research aims to deepen the understanding of the relationship between sexual satisfaction and mental health and the moderating effect of romantic partner status and gender on this relationship in adolescents and young adults; however, certain limitations must be taken into account. Furthermore, certain future lines of research are proposed as a means of addressing the persisting uncertainties regarding the sexuality of adolescents and young people. Given the cross-sectional nature of the survey and our reliance on self-reporting, responses are subject to recall bias. To minimize this effect, the participants were asked to only consider the previous month when answering. Likewise, the cross-sectional methodology does not allow for causal conclusions to be made and thus, it is strongly recommended that longitudinal studies be developed in the future. This is especially important in this area of research due to the ongoing debate about the directionality of the relationship between sexual satisfaction and mental health. We have theoretically supported one direction, but other authors defend the contrary. It is important to recognize each position on this current and unresolved debate and develop longitudinal studies and statistical analyses that shed light on this issue.

Furthermore, more information about participants’ past sexual life (i.e., sexual and romantic relationships) is relevant for a more in-depth comprehension of the results; thus, this issue must be addressed in future works. In addition, although our goal was to explore the subjective perception of sexual satisfaction and its association with mental health, more information about current relationships (i.e., sexual and romantic), such as frequency of sexual relations, could be instrumental in providing a more accurate interpretation of the discrepancies observed in sexual satisfaction. To be precise, more longitudinal research is needed to investigate how trajectories of romantic and sexual development (taking into account different behaviors and different sexual identities) run parallel to one another and how various stages and events in these trajectories are intertwined. These results could also appeal to different institutions that work with young people (e.g., schools, health centers, social services, etc.) and be incorporated in the development of future research studies on the positive aspects of adolescent and young adult sexuality. Nonetheless, one of the main goals of this study was to analyze the moderation effect of current relationship status as opposed to sexual life as a whole. 

Another limitation is that the present study evaluated symptoms of anxiety and depression but failed to evaluate differences among conditions that might conceivably be diagnosed, including those which could be medically treated; individuals with clinically diagnosed mental disorders may be more vulnerable to the consequences of poor sexual satisfaction. 

Additionally, it would be relevant to develop further research regarding sexual satisfaction in romantic relationships. First, it would be interesting to study sexual satisfaction and its predictors in more detail using the Interpersonal Exchange Model as a basis, and to test whether the difference between cost and reward accounts for anxiety and depression [70]. Additionally, future research should further examine the relationship between specific romantic and sexual aspects and sexual satisfaction and mental health. It would be of value, for example, to identify which interpersonal processes (e.g., intimacy) and cognitive assessments (e.g., guilt) of adolescent and young adult romantic relationships are likely to influence the relationship between sexual satisfaction and mental health as a protective factor. Finally, with regard to the proposed objectives, this study was carried out in a specific cultural context (Spain) with two defined age groups (adolescents and young adults), which represents a limitation when generalizing the results across cultures and age groups. Moreover, differences in the age frameworks used in the research may limit the generalization of the results. Future research should similarly replicate these findings with additional age groups using the same age frameworks, yet under different cultural contexts; moreover, it would be useful to undertake future research that would broaden knowledge about lesbian, gay, bisexual, transgender, and intersex (LGBTI+) people.

### 4.2. Practical Implications

These findings identify specific areas of intervention that may be used by practitioners who seek to support adolescents and young adults, as well as those who are dedicated to promoting healthy romantic relationships. Sexuality and mental health are still considered taboo subjects, especially among adolescents and young adults. These results may be useful for professionals concerned with wellness and protective factors (i.e., healthy romantic relationships) when seeking to encourage young people to engage solely in those romantic and sexual relationships which are satisfying. Furthermore, taking into account that most youth see a health provider at least once a year [71], medical professionals have unique opportunities to explore sexuality (although many medical providers may feel uncomfortable asking about sexuality), identify mental health problems, and help youth in gaining appropriate access to health care. 

Practitioners, as well as social workers, school psychologists, and school medicine specialists, should be trained to tackle these issues and employ intervention strategies that encourage adolescents and young adults to process negative feelings (e.g., guilt or shame), thereby helping them to recognize the socially embedded nature of these experiences. When it comes to teaching sexuality, language which can induce certain emotions must be avoided. The perception of sexuality from a purely risk perspective must be replaced by one that embraces the development of sexuality as an appropriate milestone [71] in which emotional meanings must be incorporated by necessity. It is therefore necessary that comprehensive sex education be implemented in schools, addressing not only the biological, but also, the emotional aspects of sexuality [12]. As an alternative approach to intervention, identifying the factors that heighten sexual satisfaction can enable more effective clinical responses. 

Furthermore, gender stereotypes associated with double standards have a number of negative consequences for both men and women, and therefore should be addressed and minimized in educational interventions. Beyond the double standard, the broadening of social expectations with regard to partners and sexual activity should be contemplated by professionals in so far as they are more pervasive for young adults than for adolescents (i.e., society expects young people to have intimate partners and sexual relations, but the same is not true for adolescents). In addition, the human development process regarding all the aspects involved (e.g., social, emotional) must be considered by professionals as a means of correctly explaining sexual behavior. In this regard, adolescence is often the stage of life at which sexual behavior begins, while early adulthood is the stage of sexual consolidation. Finally, practitioners also play a part in the social context in that their role is not only significant in terms of specific interventions, but also because they are key intermediaries in terms of networking with other agents. These outcomes could facilitate the development and improvement of clinical interventions, in particular factors related to sexual satisfaction and, consequently, mental health.

## 5. Conclusions

In conclusion, despite such limitations, this research is significant for several reasons. Firstly, this study draws attention to the perception of sexual satisfaction among adolescent and young adults. Although sexuality can be perceived as a controversial topic (especially adolescent sexuality), sex education is the only way to prepare young people for making healthy decisions. In addition, the literature available on the development of sexual and romantic relationships in adolescence and young adulthood focuses either on the first age group or on the second one, though they are rarely addressed collectively. Secondly, our work sheds light on how sexual satisfaction affects mental health in adolescents and young adults, while taking into account current relationship status (i.e., being in a romantic relationship or not). Previously the relationship between these variables and their moderating effects were not studied in much depth; in this sense, studies usually focused only on adolescents, college students, or adults [19]. Thirdly, the research presented here highlights the important role that romantic relationships can play in the mental health of adolescents and young adults; previous research focused primarily on individuals engaged in a romantic relationship [72], but not on the role that relationship status played. If certain moderating factors are not considered, variability between individuals could potentially mask the relationship that exists between certain variables. Furthermore, this study is focused on sexuality as a positive dimension; most studies have examined risk factors for specific sexual problems [73,74] while ignoring other positive aspects, such as sexual satisfaction. Finally, in order to clearly identify the characteristics of these variables, certain associations were established as a means of better defining strategies for the prevention of mental health problems and the promotion of quality sexual education.

## Figures and Tables

**Table 1:** Univariate ANOVA results for differences in sexual satisfaction and mental health among sexual activity level and partner status groups for adolescents and young adults.

Variables	Groups	Adolescents				Young Adults			
		Mean	SE	F	η_p_^2^	Mean	SE	F	η_p_^2^
Sexual satisfaction	Gender			2.93	0.004			0.82	0.001
	Men	3.38	0.07			3.57	0.05		
	Women	3.23	0.05			3.51	0.04		
	Partner status			168.68 ***	0.173			402.77 ***	0.317
	No partner	2.73	0.04			2.87	0.04		
	Partner	3.88	0.08			4.20	0.05		
	Gender × Partner st.			0.46	0.000			0.54	0.001
	Men without partner	2.82	0.06			2.92	0.06		
	Men with partner	3.95	0.12			4.21	0.08		
	Women without partner	2.65	0.06			2.81	0.06		
	Women with partner	3.82	0.09			4.20	0.06		
Anxiety	Gender			17.68 ***	0.021			21.11 ***	0.024
	Men	1.99	0.05			1.85	0.04		
	Women	2.28	0.04			2.08	0.03		
	Partner status			2.05	0.003			6.71 **	0.008
	No partner	2.09	0.03			2.03	0.03		
	Partner	2.18	0.06			1.90	0.04		
	Gender × Partner st.			0.248	0.000			2.61	0.003
	Men without partner	1.93	0.05			1.95	0.05		
	Men with partner	2.06	0.09			1.74	0.06		
	Women without partner	2.24	0.05			2.10	0.05		
	Women with partner	2.31	0.07			2.06	0.05		
Depression	Gender			29.88 ***	0.036			53.17 ***	0.058
	Men	2.01	0.05			1.94	0.04		
	Women	2.37	0.04			2.31	0.03		
	Partner status			0.03	0.000			8.35 **	0.010
	No partner	2.19	0.03			2.20	0.03		
	Partner	2.20	0.06			2.05	0.04		
	Gender × Partner st.			1.59	0.002			7.35 **	0.008
	Men without partner	1.96	0.05			2.08	0.05		
	Men with partner	2.06	0.09			1.80	0.06		
	Women without partner	2.41	0.05			2.32	0.05		
	Women with partner	2.34	0.07			2.31	0.05		

** *p* < 0.01; *** *p* < 0.001. SE: Standard error. st.: status.

**Table 2:** Regression models with the macro PROCESS including sexual satisfaction as a predictor of anxiety and depression, and gender and relationship status as moderators for adolescents and young adults.

	Adolescents						Young Adults					
	Anxiety			Depression			Anxiety			Depression		
	Δ*R*^2^	*B*	95% CI	Δ*R*^2^	*B*	95% CI	Δ*R*^2^	*B*	95% CI	Δ*R*^2^	*B*	95% CI
Step 1	0.037 ***			0.061 ***			0.045 ***			0.111***		
Sexual satisfaction		0.05	(−0.11, −0.21)		0.02	(−0.14, 0.18)		−0.07	(−0.22, 0.07)		−0.23 **	(−0.37, −0.09)
Gender		0.31	(−0.01, 0.62)		0.48 **	(0.17, 0.79)		0.25	(−0.05, 0.56)		0.17	(−0.13, 0.47)
Relationship status		0.64 **	(0.16, 1.11)		0.38	(−0.09, 0.84)			(−0.15, 0.72)		0.68 **	(0.21, 1.06)
												
Step 2	0.007 *			0.003			0.002			0.007 *		
Sexual satisfaction × Gender	0.000	−0.01	(−0.10, 0.09)	0.000	−0.03	(−0.12, 0.7)	0.000	−0.01	(−0.10, 0.07)	0.001	0.05	(−0.04, 0.13)
Sexual satisfaction × Relation st.	0.007 *	−0.16*	(−0.28, −0.03)	0.003	−0.09	(−0.22, 0.03)	0.002	−0.07	(−0.18, 0.04)	0.006 *	−0.13 *	(−0.24, −0.03)
												
Total *R*^2^	0.044 ***			0.064 ***			0.047 ***			0.118 ***		
*n*	809			809			873			873		

* *p* < 0.05; ** *p* < 0.01; *** *p* < 0.001.
